# Repeated salivary daytime cortisol and onset of mood episodes in offspring of bipolar parents

**DOI:** 10.1186/s40345-016-0053-5

**Published:** 2016-05-26

**Authors:** Sarah M. Goodday, Julie Horrocks, Charles Keown-Stoneman, Paul Grof, Anne Duffy

**Affiliations:** Department of Epidemiology, Dalla Lana School of Public Health, University of Toronto, 155 College St, Toronto, ON M5T 3M7 Canada; Department of Mathematics & Statistics, University of Guelph, Guelph, ON Canada; Department of Psychiatry, University of Toronto, Toronto, ON Canada; Mood Disorders Centre of Ottawa, Ottawa University Health Services, Ottawa, ON Canada; Department of Psychiatry, University of Calgary, Calgary, AB Canada

**Keywords:** Hypothalamic pituitary adrenal axis, Cortisol, Mood disorders, Bipolar disorder, High-risk offspring, Pathophysiology

## Abstract

**Background:**

Differences in cortisol secretion may differentiate individuals at high compared to low genetic risk for bipolar disorder (BD) and predict the onset or recurrence of mood episodes. The objectives of this study were to determine if salivary cortisol measures are: (1) different in high-risk offspring of parents with BD (HR) compared to control offspring of unaffected parents (C), (2) stable over time, (3) associated with the development of mood episode onset/recurrence, and (4) influenced by comorbid complications.

**Methods:**

Fifty-three HR and 22 C completed salivary cortisol sampling annually for up to 4 years in conjunction with semi-structured clinical interviews. The cortisol awakening response (CAR), daytime cortisol [area under the curve (AUC)], and evening cortisol (8:00 p.m.) were calculated.

**Results:**

There were no differences in baseline CAR, AUC and evening cortisol between HR and C (*p* = 0.38, *p* = 0.30 and *p* = 0.84), respectively. CAR, AUC and evening cortisol were stable over yearly assessments in HR, while in Cs, evening cortisol increased over time (*p* = 0.008), and CAR and AUC remained stable. In HR, AUC and evening cortisol increased the hazard of a new onset mood disorder/recurrence by 2.7 times (*p* = 0.01), and 3.5 times (*p* = 0.01), respectively, but this was no longer significant after accounting for multiple comparisons.

**Conclusions:**

Salivary cortisol is stable over time within HR offspring. However, between individuals, basal salivary cortisol is highly variable. More research is needed, with larger samples of prospectively studied HR youth using a more reliable method of cortisol measurement, to determine the potential role of cortisol in the development of mood disorders.

**Electronic supplementary material:**

The online version of this article (doi:10.1186/s40345-016-0053-5) contains supplementary material, which is available to authorized users.

## Background

Bipolar disorder (BD) is a complex psychiatric disorder that runs in families and is associated with high individual and societal burden (Judd et al. 2005; Kozloff et al. 2010). Afflicted individuals often endure years of impairing illness before receiving the appropriate diagnosis (Hirschfeld et al. 2003) and among individuals at genetic risk, non-specific manifestations develop early in adolescence (Duffy et al. 2014; Keown-Stoneman et al. 2015). Delayed diagnosis, and sub-optimal treatment contribute to the substantial burden of illness and poor quality of remission (Hirschfeld et al. 2003; Schaffer et al. 2006). The high heritability of BD is well established; however, identified susceptibility genes have so far explained only a small proportion of the risk (McGuffin et al. 2003; Farmer et al. 2007; Gottesman et al. 2010). The identification of reliable risk indicators and neurobiological targets for intervention in BD is a priority with widespread implications for improving earlier accurate detection and developing specific preventive measures.

Hypothalamic–pituitary–adrenal (HPA) axis dysregulation is the most consistent biological predictor of mood episode recurrence in both BD and melancholic depressed patients (Carroll et al. 1981; Duffy et al. 2012), while non-suppression to dexamethasone challenge has been used as a barometer for illness activity (Deshauer et al. 1999; Stetler and Miller 2011). The HPA axis is central in controlling reactions to stress and regulating emotional processes and there is an established association between mood symptoms and hypercortisolaemic states providing strong evidence of the biological plausibility of this association (Duffy et al. 2012).

The magnitude of association between basal cortisol and mood episodes is small to moderate, reflecting heterogeneity of clinical subtypes, medication, age, time of day, and endocrine methods, making studies difficult to compare and interpret. A meta-analysis of studies examining basal and post-challenge HPA axis measures suggests that major depression is associated with only a small to moderate increase in basal cortisol and adrenocorticotropic hormone (ACTH), and a decrease in corticotrophin-releasing hormone (CRH) levels among studies taking into account medication use, diagnostic heterogeneity, and age; while studies not taking these factors into account reported stronger associations between HPA axis function and depression (Stetler and Miller 2011).

There are few published longitudinal studies examining whether elevated cortisol is associated with the subsequent onset of mood disorders in offspring at high-risk for BD. The majority of research has been completed in older patients with established illness, and as such is limited by burden of illness confounds. As individuals age, the cumulative effect of acute psychiatric episodes, residual symptoms, and treatment effects over the life span makes the association between cortisol and subsequent mood episodes difficult to disentangle.

A small number of studies have reported elevated morning salivary cortisol (Goodyer et al. 2000; Halligan et al. 2004, 2007; Mannie et al. 2007) and evening urinary cortisol (Rao et al. 2009) in unaffected and remitted offspring of depressed parents compared to offspring of well parents and a longitudinal association with subsequent depressive symptoms; however, other studies have not replicated this finding including a large prospective cohort study taking into account a number of important confounding factors (Carnegie et al. 2014). Of note, some of these studies did not report a significant difference in evening salivary cortisol between offspring at high risk for unipolar depression compared to control offspring of unaffected parents (Halligan et al. 2004; Rao et al. 2009). Ellenbogen et al. published a number of studies demonstrating evidence of higher mean cortisol awakening response (CAR) and daily cortisol secretion as measured by area under the curve (AUC) among the adolescent offspring of parents with BD compared to well parents (Ellenbogen et al. 2004; Ellenbogen et al. 2006). However, this finding was not replicated in the same sample at an older age, although high-risk offspring still exhibited higher afternoon salivary cortisol over a two-week period compared to controls (Ellenbogen et al. 2010) and there was evidence that morning cortisol predicted the development of major mood episodes (Ellenbogen et al. 2011). In a small pilot study using the Flourish Canadian high-risk cohort, there was evidence of an elevated cortisol awakening response in remitted BD patients and in the first 30 min after awakening in their high-risk offspring (Deshauer et al. 2003, 2006). There is also preliminary evidence of differences in neurotrophic and immunological markers between high-risk offspring of a bipolar parent and control offspring of well parents and in the early compared to later stages of illness development in high-risk offspring (Duffy et al. 2014). Differences in basal cortisol among high-risk offspring compared to controls and the association with major mood episodes in high-risk offspring suggest that differences in HPA axis indices may reflect a genetic vulnerability associated with BD. However, the findings to date are mixed, and further replication in high-risk samples is necessary to confirm this theory.

The objectives of this paper are to: (1) determine if there are differences in salivary cortisol in offspring of parents with BD compared to offspring of unaffected parents, (2) describe the stability of salivary cortisol measures within high-risk and control offspring over several years, (3) determine the longitudinal association between salivary cortisol and risk of mood episodes in high-risk offspring and (4) explore the influence of frequent complications of BD, including psychotic symptoms and substance use disorders (SUD), on the association between cortisol and risk of mood disorder in high-risk offspring. We hypothesized that indices of daytime cortisol secretion (AUC), morning awakening response (CAR) and evening salivary cortisol, would be elevated and less stable in high-risk compared to control offspring, and that psychotic symptoms and SUD would be an effect modifier.

## Methods

### Participants

Participants were a subset of high-risk and control offspring from the Flourish Canadian Offspring Cohort Study, which is an ongoing, dynamic, prospective cohort study based in Ontario that began in 1996. Descriptions of this study cohort are given in detail elsewhere (Duffy et al. 2009, 2010, 2014). Briefly, offspring were considered eligible for recruitment into the study if they were between the ages of five and 25 years. High-risk offspring had one parent with confirmed BD I or II at baseline and no other major psychiatric comorbidity. Their other biological parent was also confirmed to have no major psychiatric disorder at baseline. Control offspring were eligible if both of their parents were confirmed to have no major psychiatric disorder (psychosis, SUD or major mood disorder) at baseline. Offspring were excluded if they were unable to comprehend or comply with the study protocol. All diagnoses in the parents were made by expert psychiatrists in accordance with DSM-IV-TR criteria following schedule for affective disorders: present and lifetime (SADS-PL) (Endicott and Spitzer 1978) interviews and were confirmed through a consensus review with at least two additional research psychiatrists blind to family affiliation. For this sub-study, we included consenting/assenting high-risk and control offspring over the age of seven years from the larger cohort. At the time of sampling, all offspring were either unaffected for lifetime mood disorder or in clinical remission based on semi-structured clinical research interview and established cutoff scores on the Beck Depression Inventory (BDI) (Beck and Beamesderfer 1974) and hypomania checklist revised (Young et al. 1978). All offspring/parents completed an informed consent/assent form. This research was approved by the local research ethics boards (Ottawa Institutional Review Board and the IWK Research Ethics Board in Halifax).

### Procedure

High-risk and control offspring completed annual clinical assessments with expert psychiatrists following Kiddie SADS-PL (Axelson et al. 2003)/SADS-PL (Endicott and Spitzer 1978) interviews and completed a self-report measure of life stress (Goodyer et al. 1990, 1997) during each annual visit. Socio-economic status (SES) was measured in the parents using the Hollingshead SES scale; a composite measure of both working spouses’ education and occupation (Hollingshead 1970). During the annual research visit, consenting offspring and their parents were given oral and written detailed instructions on the salivary cortisol collection protocol. Saliva was collected using five-ml free drool Sarstedt plastic cryovials. On three consecutive routine weekdays, offspring provided salivary samples four times a day, upon awakening (T0), 30 min later (T1), 60 min later (T2) and at 8:00 p.m. (T3). Offspring were instructed to not brush their teeth, smoke, eat or drink anything with the exception of water before their T0, T1 and T2 samples, and 1 h before their T3 sample. Offspring were also instructed not to perform any vigorous exercise one hour prior to their salivary samples and not drink alcohol during their three sampling days. Offspring recorded their date and time of sampling for every sample, their sleep quality and ease of waking quality on each consecutive sampling day, and their mood symptoms (Kent et al. 1997) on one of the three consecutive sampling days. Consenting offspring repeated the 3-day cortisol assessment after their subsequent annual clinical interviews. The range of repeated assessments after yearly clinical interviews was 2.0–3.5 years in high-risk and 0.9–3.0 years in control offspring. Samples were kept frozen in the subjects’ own freezers and transported on ice to the laboratory. Salivary cortisol levels were measured in batches (by year) using an enzyme immunoassay kit according to the kit instructions (Salimetrics, State College, PA, USA). The intra-assay coefficient of variation was 2.1 %. Four batches were analyzed and the inter-assay coefficient of variation (using different reference sample concentrations) was 1.9, 0.0, 3.9 and 3.0 %. The minimum detectable concentration of cortisol for all batches was 0.33 nmol/L.

### Measures

Salivary cortisol levels were averaged over the three consecutive sampling days at each time point for each sampling year to account for day to day variation. To replicate findings from other studies, we were interested in measuring the rise in cortisol upon awakening, an integrated index of daytime secretion of cortisol, and evening cortisol. To measure these indices of HPA axis activity, we calculated the mean CAR (T1 minus T0), mean area under the curve ground (AUC) (T0, T1 and T3), and mean T3 (8:00 p.m.) cortisol, respectively. Owing to considerable missing data at 60 min (T2), CAR was calculated using only the second morning sample (T1: 30 min post-awakening). We were confident that we were able to capture the awakening response using 30 min post-awakening, and there is evidence of similar cortisol concentrations at 30 and 60 min post-awakening in patients with major depression (McAllister-Williams et al. 2015). Daytime secretion was measured by AUC ground (Pruessner et al. 2003), comprising the total area under the absolute cortisol measurements at T0, T1 and T3. Evening cortisol was measured by the mean of the three evening cortisol samples at 8:00 p.m. (T3). Life stress was measured using the recent life events and difficulties questionnaire (LEQ) (Goodyer et al. 1990, 1997) during annual clinical interviews and mood symptoms were measured during one of the three consecutive sampling days of the salivary cortisol collection using the Mood and Feelings Questionnaire (MFQ) (Kent et al. 1997). The LEQ and MFQ both have established reliability and validity in this population (Goodyer et al. 1990; Daviss et al. 2006). Sleep quality and ease of waking during the sampling collection were captured using the subjective sleep rating scale (Bertocci et al. 2005); a seven-item self-report shortened measure of the Pittsburgh sleep index (Buysse et al. 1989). For the sleep items, participants rated their response on a 100-point scale ranging from 0 (very bad, very difficult) to 100 (very good, very easy). The sleep ratings were averaged over the three day sampling period. Pubertal status was collected at the time of sample collection using the Peterson Pubertal Development Scale (PPDS) (Petersen et al. 1988). The PPDS has demonstrated good reliability and validity (Petersen et al. 1988).

Lifetime psychotic symptoms and/or SUD were captured during the annual KSADS-PL/SADSL-PL clinical interviews. Lifetime psychotic symptoms included symptoms that occurred either inside or outside (psychosis not otherwise specified) of mood episodes. We intended on including lifetime hospitalizations; however, only one participant from this analysis was hospitalized. The offspring in this analysis were still in the early stages of illness development, and therefore had minimal history of pharmacological treatment (Duffy et al. 2014).

### Data analysis

Cox proportional hazard models, with sandwich estimators to account for sibling correlation (clustering within families) and time-varying covariates to account for sampling year, were estimated to determine the association between the pattern of cortisol values over time and the development of a new onset mood disorder and major mood episode recurrences (hypomania, mania, major depression) over follow-up from date of birth. All survival models were checked for proportionality. Date of birth was used rather than date of baseline interview as this study uses an open, dynamic cohort design where subjects enter the study at different ages and dates. Date of last interview was used as a censoring variable. Mixed models with random intercepts to account for sibling correlation were used to test for differences in baseline cortisol measures between high-risk and control offspring. Mixed models were also used to determine the stability of cortisol measurements over repeated sampling years using an autoregressive correlation structure, accounting for correlation among measurements taken on the same individual over multiple years. Independent interaction terms were included in the mixed model to determine if complications (i.e., psychotic symptoms × sampling year and SUD × sampling year) influenced the stability of cortisol over time. Cortisol levels pertaining to AUC and evening cortisol (T3) were log transformed using the natural logarithm to accommodate the normality assumption of all mixed models. Normality of the outcome is not a required assumption for Cox proportional hazard models, and therefore cortisol levels pertaining to these models were untransformed.

All models were adjusted for age of offspring at baseline cortisol sample, and sibling correlation. Models pertaining to high-risk offspring only were additionally adjusted for sex. Multiple comparisons were accounted for using the Šidák correction for multiple comparisons.

To minimize the impact of missing data, each subject’s cortisol measurements at T0, T1, T2 and T3 were averaged over the three collection days within each year for the four separate years per subject. This yielded an average T0, T1, T2 and T3 cortisol value for each repeated annual assessment. For example, if a subject had missing data for one day, the average was calculated using the two other available days. Any subjects that were missing cortisol measurements on all three days were omitted from the analysis.

## Results

### Baseline characteristics

Fifty-three high-risk and 22 control offspring completed the baseline (year 1) cortisol sampling study. High-risk and control offspring did not differ significantly on baseline characteristics except for body mass index (BMI), where BMI was higher in controls (*p* = 0.01) (Table 1). Sixty-eight percent of high-risk offspring and 55 % of controls were female (*p* = 0.27). All control offspring and 77 % of HR offspring had completed puberty at baseline or were in Tanner pubertal stage IV. The mean age at cortisol sampling in year 1 was 20.0 years (standard deviation (SD) = 7.2, range = 11.3–33.1) in high-risk and 20.8 years (SD = 2.8) in control offspring. Seventeen (32 %) high-risk offspring had a lithium-responsive parent while 36 (68 %) had a lithium non-responsive parent. The median and range of sample times across repeated sampling years can be found in Additional file 1: Table S1.

**Table 1:** Baseline characteristics of high-risk and control offspring

	High-risk *N* = 53	Control *N* = 22	*p* value
Sex *n* (%)			
Male	17 (32.08 %)	10 (45.45 %)	0.2718^a^
Female	36 (67.92 %)	12 (54.55 %)	
SES^d^ *n* (%)			
1 (low)	0	0	0.3404^a^
2	1 (1.89 %)	1 (4.55 %)	
3	1 (1.89 %)	2 (9.09 %)	
4	24 (45.28 %)	8 (36.36 %)	
5 (high)	27 (50.94 %)	11 (50.00 %)	
Pubertal status^e^ *n* (%)			
1 (pre)	1 (2.08 %)	0	0.2045^a^
2	2 (4.17 %)	0	
3	5 (10.42 %)	0	
4	11 (22.92 %)	1 (6.25 %)	
5 (post)	29 (60.42 %)	15 (93.75 %)	
Body mass index mean (SD)	22.43 (4.50)	25.57 (3.60)	0.0122^b^
Age at baseline sample collection, mean (SD)	20.00 (7.23)	20.76 (2.76)	0.9632^b^
Mean MFQ score (SD)	10.60 (8.50)	6.20 (9.00)	0.0631^b^
Mean sleep quality^f^ (SD)	75.18 (13.75)	76.00 (12.87)	0.8210^b^
# of undesirable life events in previous year^g^ *n* (%)	1.09 (1.20)	0.83 (0.99)	0.3626^c^

*SD* standard deviation, *SES* socio-economic status, *MFQ* mood and feelings questionnaire

^a^Fisher’s exact test or Chi square

^b^ *T* tests

^c^Poisson regression

^d^Hollingshead SES scale

^e^Peterson pubertal scale

^f^Pittsburgh sleep index

^g^Recent life events and difficulties questionnaire

### Follow-up

Thirty-two high-risk offspring completed a second repeat cortisol measurement after their subsequent annual research visit, 21 completed a third repeat cortisol measurement and six a fourth repeat cortisol measurement. In the controls, eight completed a second repeat cortisol measurement, and two completed a third repeat measurement. No controls completed a fourth repeat measurement. In total, 60 % (32/53) of high-risk offspring did not complete up to three repeated assessments, while very few control offspring completed up to three repeated assessments. Main reasons for attrition were non-compliance: offspring found the protocol demanding and many did not continue for this reason. Further, several offspring moved away as many were at the age of starting university, and therefore the protocol could not be continued. Sensitivity analyses were conducted: the offspring dropping out at the year-two assessment were not significantly different from offspring completing all yearly assessments in age or lifetime mood diagnosis (all *p* > 0.05). In high-risk and control offspring, there were more males in the dropout group compared to the sample of offspring who completed all assessments (41 vs. 26 % and 29 vs. 47 %, respectively). Owing to the small numbers of yearly repeat assessments in the controls, primary analyses with the control group are limited to baseline cortisol measures.

Potential confounders were explored, including age, sex, BMI, SES, mean sleep quality of the night preceding the sampling day, number of undesirable life events in the preceding year and awakening time when baseline cortisol sample was taken. There was no significant association between any of the potential confounding factors and cortisol measured by CAR, logAUC, and log evening cortisol (all *p* > 0.05).

Among the 53 high-risk offspring, 14 (26 %) developed a DSM-IV new onset mood episode (*n* = 9) or recurrence (*n* = 5) over study follow-up. In total, 26 (49 %) high-risk offspring were diagnosed with a DSM-IV lifetime mood disorder (major depression (MD), BD I, II, not otherwise specified (NOS), dysthymia, depression NOS) but 17 (32 %) of these cases developed prior to baseline. Five high-risk offspring had lifetime psychotic features and seven had a lifetime SUD. No control offspring developed diagnosable psychopathology over follow-up or had psychopathology prior to baseline.

### Between subjects differences (high risk vs. control)

**Fig. 1:**
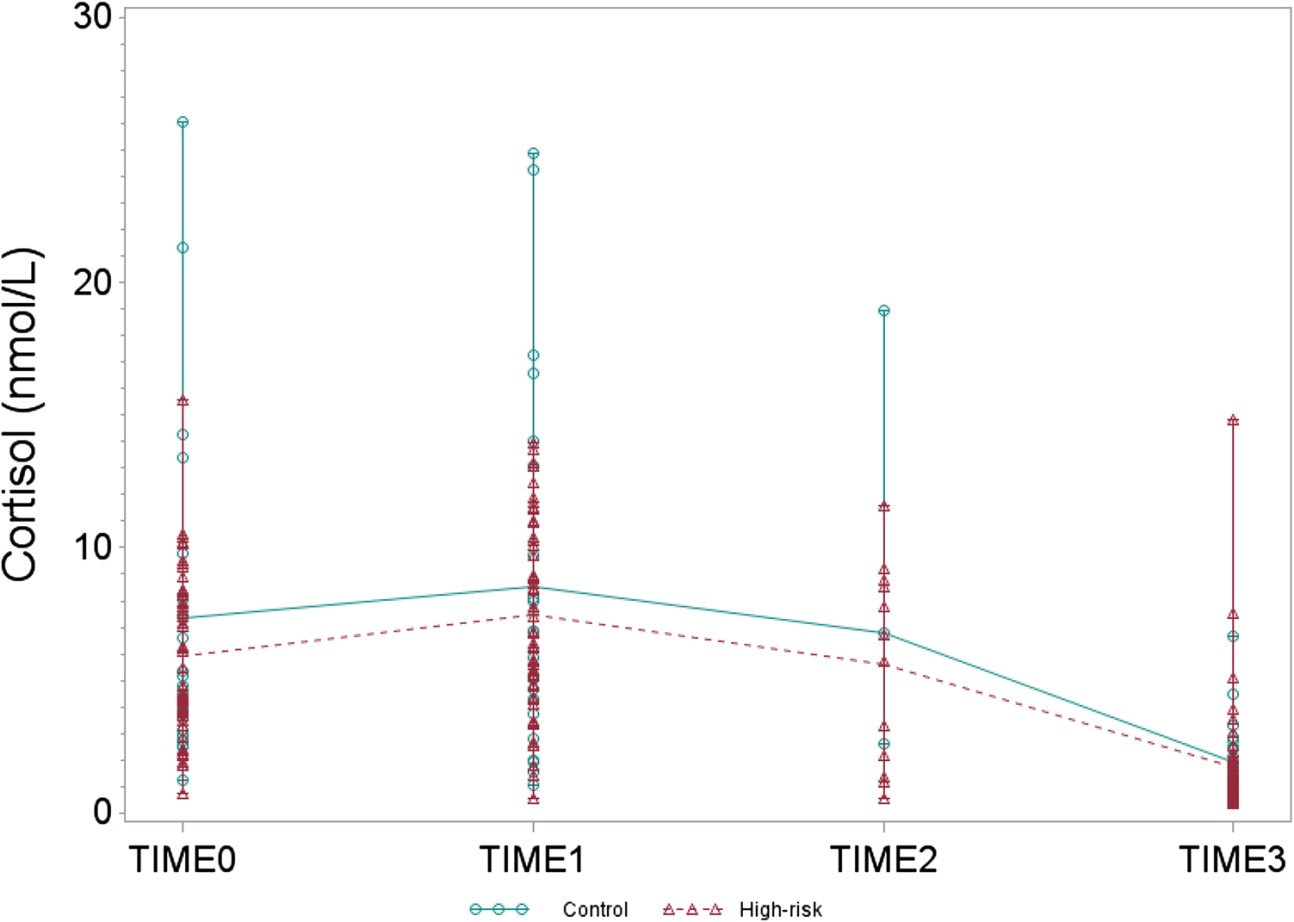
3-day mean baseline saliva cortisol levels (nmol/L) at awakening (Time 0), 30 min later (Time 1), 60 min later (Time 2) and at 8:00 p.m. (Time 3) in high-risk and control offspring. Sample size in high-risk group at Time 0: 53; Time 1: 53; Time 2: 12; Time 3: 53 and sample size in control group at Time 0: 22; Time 1: 22; Time 2: 3; Time 3: 21

Cortisol levels at baseline were highly variable in high-risk and control offspring (Table 2). Mean baseline salivary cortisol concentrations at all time points during the day did not differ between high-risk and control offspring (Fig. 1). Moreover, using adjusted mixed models, baseline CAR, logT3, and logAUC were not significantly different between high-risk and control offspring (*p* = 0.38, *p* = 0.30 and *p* = 0.84), respectively (Additional file 1: Table S2).

**Table 2:** Mean baseline cortisol values (nmol/L) in high-risk and control offspring

Cortisol measurement	High-risk			High-risk new mood episode/recurrence			High-risk unaffected			Control		
	Mean	SD	*n*	Mean	SD	*n*	Mean	SD	*n*	Mean	SD	*n*
CAR	1.5821	2.6985	53	1.1117	2.3158	14	1.4511	2.7074	27	1.1883	4.0286	22
Time 3^a^	1.7211	2.2336	53	2.1078	1.7483	14	1.7710	2.8267	27	1.9424	1.5352	21
AUC^b^	212,337.09	88,505.58	47	216,718.2277	92,904.24	14	206,593.52	99,296.26	23	265,085.77	185,938.31	19

Values are untransformed and unadjusted

*SD* standard deviation, *CAR* cortisol awakening response, *AUC* area under the curve (ground)

^a^8:00 p.m. cortisol

^b^Seconds*nmol/L

### Stability of cortisol concentrations over time

**Fig. 2:**
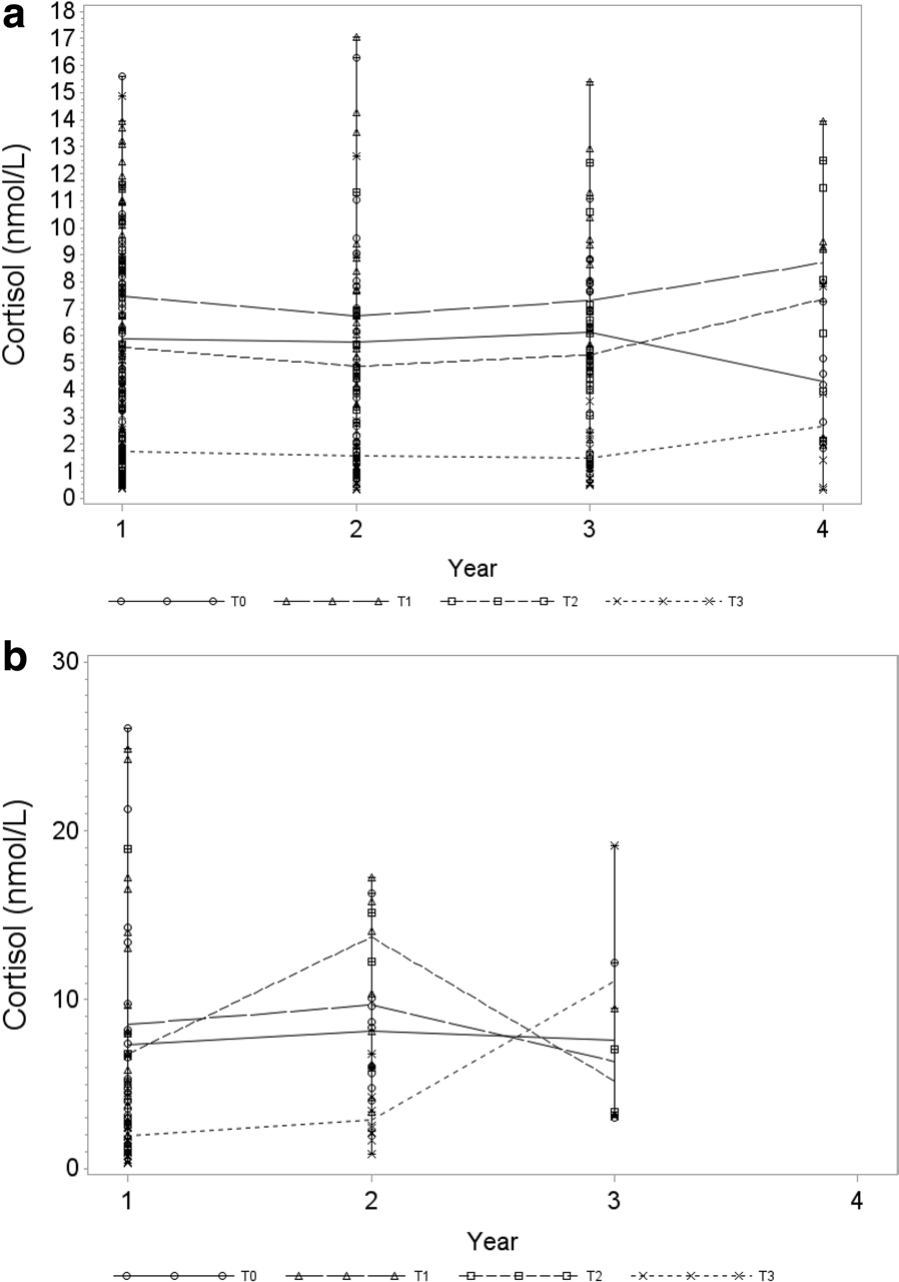
**a** 3-day mean saliva cortisol levels at awakening (T0), 30 min later (T1), 60 min later (T2) and at 8:00 p.m. (T3) across repeated sampling years in high-risk offspring. **b** 3-day mean saliva cortisol levels at awakening (T0), 30 min later (T1), 60 min later (T2) and at 8:00 p.m. (T3) across repeated sampling years in control offspring

Unadjusted cortisol levels across repeated sampling years in high-risk and control offspring can be found in Fig. 2. Using adjusted mixed models, in high-risk offspring, there was no significant difference in cortisol levels across repeated sampling years (CAR: *p* = 0.45, logAUC: *p* = 0.40, logT3: *p* = 0.66). In controls, logT3 cortisol significantly increased over repeated sampling years (*p* = 0.0077), while all other measurements of cortisol were stable over time (CAR: *p* = 0.25, logAUC: *p* = 0.86).

### Within subject differences (high risk only)

Mean baseline cortisol levels within high-risk offspring can be found in Table 2. Mean baseline evening cortisol was higher among high-risk offspring with a new onset mood episode or recurrence (2.1 nmol/L, SD = 1.7) compared to unaffected high-risk offspring (1.8 nmol/L, SD = 1.8) (Table 2), although this was not statistically significant (*p* > 0.05).

To determine if cortisol levels predicted a new onset mood episode or recurrence among high-risk offspring (*n* = 14/53) over time, a longitudinal mixed model was computed accounting for annual repeated cortisol measurements. Before adjustment for multiple comparisons, the pattern of logAUC and logT3 over repeated assessment times significantly increased the adjusted hazard of a new onset mood episode or recurrence by 2.7 times (*p* = 0.01, 95 % CI 1.2, 5.8), and 3.5 times (*p* = 0.01, 95 % CI 1.3, 9.4), respectively. However, after correction for multiple adjustments, these associations were no longer statistically significant (Šidák correction cutoff *p* = 0.0043). The pattern of CAR over repeated assessment times was not associated with the hazard of a new onset mood episode or recurrence over follow-up (HR 0.9, *p* = 0.19, 95 % CI 0.7, 1.1).

### The influence of clinical characteristics

Using adjusted mixed models, in high-risk offspring with a lifetime mood disorder (*n* = 26/53), clinical characteristics including lifetime psychotic symptoms, comorbid SUD, or parent lithium response did not significantly predict baseline CAR, logAUC, or logT3 cortisol (all *p* > 0.05) (Additional file 1: Table S3). There was no significant interaction or main effect of offspring sex with psychotic symptoms, comorbid SUD and parent lithium response predicting all three baseline cortisol measurements (all *p* > 0.05).

Using adjusted mixed models and accounting for multiple comparisons, in high-risk offspring with a lifetime mood disorder, there was no significant evidence that SUD or lifetime psychotic symptoms influenced the stability of CAR or logT3 over multiple years as evidenced by non-significant interaction terms (*p* > 0.05) (Table 3). Before adjustment for multiple comparisons, lifetime psychotic symptoms significantly interacted with cortisol sampling year to predict logAUC (*p* < 0.0001). Specifically, logAUC decreased over each sampling year from baseline among high-risk offspring with lifetime psychotic symptoms, but remained stable over time among those without lifetime psychotic symptoms. Before adjustment for multiple comparisons, there was also a significant interaction between SUD and cortisol sampling year predicting logAUC (*p* = 0.03) (Table 3). After applying a multiple comparisons correction, the only interaction that remained significant was between lifetime psychotic symptoms and logAUC (*p* = 0.0001).

**Table 3:** The influence of DSM-IV substance use disorders and lifetime psychotic symptoms on the stability of cortisol (nmol/L) in high-risk offspring over repeated annual sampling assessments

Predictor	Main effect	Sampling year interaction^b^ *p* value^c^	Main effect without interaction^b^ *p* value^c^	Number of longitudinal observations
CAR	SUD	0.1733	0.1148	101
	Psychotic	0.1687	0.1198	101
logTime 3^d^	SUD	0.1633	0.8117	102
	Psychotic	0.2728	0.0191^a^	102
logAUC^e^	SUD	0.0346^a^	0.1831	95
	Psychotic	*<0.0001*	0.4902	95

Italic denotes significance after correction for multiple comparisons

*SUD* substance use disorders, *CAR* cortisol awakening response, *AUC* area under the curve (ground), *Psychotic* lifetime psychotic symptoms in or outside mood episodes

^a^ *p* value not significant after Šidák correction for multiple comparisons (*p* value cutoff of 0.0034)

^b^Interaction term was composed of sampling assessment year and presence of lifetime SUD or psychotic symptoms

^c^Mixed model accounting for sibling correlation adjusted for sex and age at baseline cortisol sample

^d^8:00 p.m. cortisol

^e^Seconds*nmol/L

There was a significant main effect of lifetime psychotic symptoms predicting logT3 cortisol (*p* = 0.04), where high-risk offspring with a mood disorder and psychotic symptoms had 0.64 nmol/L units higher logT3 cortisol compared to high-risk offspring with a mood disorder without psychotic symptoms after accounting for sampling year, age at baseline and sex. However, this effect was no longer significant when applying a multiple comparison correction (Table 3).

## Discussion

This study examined the longitudinal association between salivary cortisol as measured by CAR, AUC and mean evening cortisol and mood episodes in offspring at high familial risk. Major findings included that salivary cortisol levels were more variable in controls compared to high-risk offspring, but did not differentiate high-risk from control offspring (Figs. 1, 2). Increasing AUC and mean evening cortisol significantly increased the hazard of a new onset or recurrence of a mood episode in high-risk offspring by 2.7 and 3.5 times, respectively; however, after correction for multiple comparisons these fell short of statistical significance.

Salivary cortisol was quite stable over the repeated sampling years in high-risk and control offspring with the exception of evening 8:00 p.m. cortisol increasing in control offspring over time. The number of controls that completed the yearly cortisol assessment post-baseline was small; therefore, the latter finding should be interpreted with caution and may reflect random error. Lifetime psychotic symptoms in high-risk offspring with a remitted mood disorder significantly influenced the stability of cortisol daily secretion over repeated sampling years as measured by AUC. In particular, a lifetime history of psychotic symptoms in high-risk offspring with a remitted mood disorder decreased salivary cortisol levels over time compared to no change among those without lifetime psychotic symptoms. In high-risk offspring with a lifetime mood disorder, a comorbid lifetime history of a SUD also influenced stability of daily secretion (AUC), where cortisol levels decreased over time among those with a prior SUD and remained stable among those without a SUD; however, the interaction pertaining to SUD did not maintain significance after adjustment for multiple comparisons. These interactions were also based on small samples sizes and, therefore, should be interpreted with caution. There were five high-risk offspring with psychotic symptoms at baseline and only one of these offspring completed a second annual repeated cortisol assessment. Seven high-risk offspring had comorbid SUD at baseline, and only two of these offspring completed three repeated annual cortisol assessments.

Our findings were not consistent with those from a similar study of offspring of parents with BD that reported differences in the CAR between high-risk and control offspring. This Montreal-based study used MEMS TrackCap technology to time stamp the collection of samples which may be more reliable than our self-report method of verifying timing of collection: although our cohort was well instructed and reported the actual sampling times. We had difficulty in compliance with our third morning sample (60 min post-awakening), which resulted in low sample sizes for this sampling time point. Several of our participants indicated that it was too difficult to complete this sample before eating breakfast and going to school or work.

The following limitations should be noted. Models pertaining to new onset mood episode and recurrences were based on small numbers making more intricate within subject analyses challenging. Future research should examine the longitudinal association between salivary cortisol indices, and the subsequent onset of mood disorder and recurrences separately. The omission of the time 2 sample (owing to low numbers) in the calculation of the AUC may possibly explain the non-significant findings as this may not have been able to effectively capture the complete rise in cortisol upon awakening. However, there is evidence of similar cortisol concentrations at 30 and 60 min post-awakening in patients with major depression (McAllister-Williams et al. 2015). The levels of cortisol in our sample were also highly variable in both high-risk and control offspring, potentially reflecting the high variability of basal salivary cortisol (Levine et al. 2007) and/or measurement error as well as the wide age range of our sample. To reduce the chance of Type II error, we had to limit the number of covariates (potential confounders) in our models. However, we explored several potential confounders and found that they were not associated with any of the cortisol measurements in high-risk and control offspring, including BMI. Moreover, models were adjusted for age and sex.

Salivary cortisol is a measure of acute change in free (unbound) cortisol and is highly sensitive to stress, daily activities, and consumption of numerous agents making compliance essential to its accurate measurement. Although the measurement of cortisol through saliva has advantages such as non-invasiveness compared to other methods (Aardal and Holm 1995), the high variability, need for a demanding collection protocol, and difficulty with reproducibility of findings appears to limit its utility in clinical practice and research. Based on this comprehensive study in well-characterized youth, we conclude that a standardized test such as the dexamethasone suppression test (DST) would be preferable to basal cortisol methods. The DST may require more invasive procedures, but it is a well-validated prognostic measure in patients with a primary recurrent mood disorder. Prospective study of the DST serially in remitted patients with lithium-responsive BD has proven clinical utility in identifying periods of heightened risk of recurrence by acting as a reliable barometer of underlying illness activity (Grof et al. 1983). This has been important, for example, in informing safe periods for planned discontinuation and for clinical monitoring to prevent relapse. Furthermore, in an unpublished clinical case series of symptomatic high-risk offspring who had not yet developed a diagnosable mood disorder (i.e., anxiety and panic disorder, circadian disturbances), the DST was uniformly positive during the acute episodes (Grof unpublished).

## Conclusions

Basal salivary cortisol is stable over time in early adulthood among high-risk offspring. Further research with larger sample sizes and more refined longitudinal methods of cortisol measurement are needed to confirm the potential role of cortisol in the development of mood disorders among individuals at genetic risk for BD. Using the DST as a measure of illness propensity and development in prospectively studied high-risk and control offspring is a promising avenue for future research given the high variability of findings pertaining to salivary cortisol across studies.

## Additional file

10.1186/s40345-016-0053-5 Mean baseline sampling times in high-risk and control offspring. **Table S2.** Baseline salivary cortisol (nmol/L) in 53 high-risk compared to 22 control offspring. **Table S3.** Clinical variables predicting baseline cortisol (nmol/L) in high-risk offspring with a DSM-IV mood disorder.

## Abbreviations

BD: bipolar disorder
HR: high risk (abstract only)
C: control (abstract only)
CAR: cortisol awakening response
AUC: area under the curve
HPA: hypothalamic pituitary adrenal
ACTH: adrenocorticotropic hormone
CHR: corticotrophin releasing hormone
SUD: substance use disorder
DSM-IV-TR: Diagnostic and Statistical Manual for Mental Disorders: 4th version—twice revised
SADS-PL: schedule for affective disorders: present and lifetime version
KSADS-PL: Kiddie Schedule for Affective Disorders: present and lifetime version
SES: socio-economic status
T0: time 0
T1: time 1
T2: time 2
T3: time 3
LEQ: Life Events Questionnaire
MFQ: Mood and Feelings Questionnaire
PDDS: Peterson Pubertal Development Scale
BMI: body mass index
MD: major depression
DST: dexamethasone suppression test
