# Supplementary material for: Repeated salivary daytime cortisol and onset of mood episodes in offspring of bipolar parents
Source: Int J Bipolar Disord. 2016 May 26;4:12. doi: 10.1186/s40345-016-0053-5 (PMC4882311; doi:10.1186/s40345-016-0053-5)
Supplement: Supplementary file 1 — 10.1186/s40345-016-0053-5 Mean baseline sampling times in high-risk and control offspring. Table S2. Baseline salivary cortisol (nmol/L) in 53 high-risk compared to 22 control offspring. Table S3. Clinical variables predicting baseline cortisol (nmol/L) in high-risk offspring with a DSM-IV mood disorder. [file 40345_2016_53_MOESM1_ESM.docx]

Table S1. Mean baseline sampling times in high-risk and control offspring

|  | High-risk | | | | Control | | | |
| --- | --- | --- | --- | --- | --- | --- | --- | --- |
|  | Time 0 | Time 1 | Time 2 | Time 3 | Time 0 | Time 1 | Time 2 | Time 3 |
| **Year 1**  Mean  (SD) | 7:20:44AM  1:09:24 | 7:53:23AM  1:08:25 | 8:33:31AM  1:16:21 | 8:31:43PM  0:52:45 | 7:33:09AM  1:25:14 | 8:08:57AM  1:10:30 | 8:55:00AM  1:04:48 | 8:12:15PM  0:57:58 |
| **Year 2**  Mean  (SD) | 7:31:42AM  1:17:10  92 | 8:10:26AM  1:24:09  92 | 9:06:29AM  1:22:15  38 | 8:22:24PM  0:54:11  86 | 7:46:35AM  1:24:15  24 | 7:17:48AM  1:26:30  24 | 8:37:40AM  2:00:04  6 | 8:07:48PM  0:55:55 |
| **Year 3**  Mean  (SD) | 7:57:41AM  1:41:04 | 8:28:32AM  1:42:47 | 9:02:31AM  1:43:17 | 8:35:17PM  0:50:47 | 8:20:20AM  1:07:03 | 8:51:30AM  1:06:41 | 9:20:50AM  1:06:40 | 8:47:30PM  0:43:54 |
| **Year 4**  Mean  (SD) | 7:54:40AM  0:58:24 | 8:25:00AM  0:58:04 | 8:55:00AM  0:58:20 | 8:20:13PM  0:47:18 | -  -  - | -  -  - | -  -  - | -  -  - |

SD: standard deviation

Table S2. Baseline salivary cortisol (nmol/L) in 53 high-risk compared

to 22 control offspring

| Cortisol measurement | Coefficient^a^ | p-value^b^ | n |
| --- | --- | --- | --- |
| CAR | 0.7767 | 0.3761 | 75 |
| logTime 3^c^ | -0.2282 | 0.2958 | 74 |
| logAUC^d^ | -0.0372 | 0.8381 | 66 |

CAR: cortisol awakening response; AUC: area under the curve (ground)

^a^Mixed model accounting for sibling correlation and age of offspring at baseline

^b^Šidák correction for multiple comparisons p-value cut off: p=0.0170

^c^8:00pm cortisol

^d^seconds*nmol/L

Table S3. Clinical variables predicting baseline cortisol (nmol/L) in high-risk offspring with a DSM-IV mood disorder

|  | p-value^a^ | | |
| --- | --- | --- | --- |
|  | CAR | LogTime 3^b^ | LogAUC |
| Psychotic | 0.2630 | 0.1600 | 0.4918 |
| SUD | 0.1202 | 0.6890 | 0.3618 |
| Lithium Responder Family | 0.7037 | 0.1556 | 0.7174 |

CAR: cortisol awakening response; AUC: area under the curve (ground); SUD: substance use disorder; Psychotic: lifetime psychotic symptoms in and outside mood episodes

^a^Mixed model adjusted for sex and age at baseline; Šidák correction for multiple comparisons p-value cutoff: p=0.0043

^b^8:00pm cortisol
